# Correlation between cow’s milk protein allergy and otitis media: a systematic review

**DOI:** 10.1016/j.bjorl.2021.07.005

**Published:** 2021-10-18

**Authors:** Karen Amanda Soares de Oliveira, Marina Tomaz Esper, Morgana Lívia de Oliveira, Marise Helena Cardoso Tofoli, Melissa Ameloti Gomes Avelino

**Affiliations:** a Universidade Federal de Goiás (UFG), Faculdade de Medicina, Goiânia, GO, Brazil; b Hospital Estadual Geral de Goiânia Dr. Alberto Rassi (HGG), Goiânia, GO, Brazil; c Hospital Materno Infantil Dr. Jurandir do Nascimento (HMI), Goiânia, GO, Brazil; d Universidade Federal de Goiás (UFG), Hospital das Clínicas (HC), Goiânia, GO, Brazil

**Keywords:** Milk hypersensitivity, Milk proteins, Otitis media, Otitis media with effusion

## Abstract

**Objectives:**

To review the evidence pertaining to the association between cow’s milk protein allergy and recurrent acute otitis media and otitis media with effusion.

**Methods:**

The CENTRAL, Web of Science, EMBASE, MEDLINE, LILACS databases, and gray literature were searched.

**Results:**

Four studies were included, identifying the prevalence rates: 0.2% of delayed speech due to chronic otitis media with effusion in 382 children with cow’s milk protein allergy, 10.7% of cow’s milk protein allergy in 242 children who underwent ENT procedures, 40% of cow’s milk protein allergy in 25 children with recurrent otitis media with effusion and higher tendency to otitis media in children with cow’s milk protein allergy of 186 children (1.5 + 0.6 *vs*. 0.4 + 0.1; *p* < 0.1).

**Conclusion:**

Considering the characteristics and methodological variations of the identified studies, it is not possible to state that there is reliable evidence of an association between cow’s milk protein allergy and otitis media.

## Highlights

• The correlation between cow’s milk protein allergy and otitis media has been investigated in recent decades.
• Allergic inflammation in the nasal mucosa is associated with Eustachian tube dysfunction, contributing to otitis media.
• Previous studies about cow’s milk protein allergy and otitis media have limitations, leading to controversial results.
• There is no reliable evidence that cow’s milk protein allergy is related to acute otitis media or otitis media with effusion.

## Introduction

Otitis media is one of the most common childhood diseases, affecting more than 90% of the pediatric population up to two years of age. It may present as acute otitis media (AOM), with rapid onset of signs and symptoms of inflammation of the middle ear mucoperiosteum, or as otitis media with effusion (OME), characterized by the presence of fluid in the tympanic cavity without signs of acute ear infection.^1^

The clinical course and sequelae of otitis media are variable; some children show spontaneous resolution without any specific treatment, while others, even after antibiotic therapy and/or surgery, develop recurrent episodes.^1^, ^2^ The identification of risk factors associated with the development of recurrent otitis media (ROM) is extremely important to identify the most susceptible children, reduce the indiscriminate use of antimicrobials and provide guidance to parents and caregivers, in the attempt to prevent auditory and/or structural sequelae.^3^

Studies indicate that at least 25% of children with OME develop a chronic condition, with the presence of fluid in the tympanic cavity for more than 3 months. Moreover, 15%–20% of children who have had AOM will have ROM.^1^, ^3^ Both may be associated with hearing loss, balance alterations, poor school performance, behavioral problems, and auditory discomfort, with a major impact on the quality of life.^4^, ^5^ Despite the large number of publications on risk factors for the development of ROM and OME, controversies remain regarding their management and specific recommendations. Thus, the search for the correlation between cow’s milk protein allergy (CMPA) and the development of otitis media is included in this context.

Cow’s milk protein is the main food allergen in children under 3 years of age and this prevalence decreases to less than 1% in children aged 6 years of and older.^6^, ^7^ However, the actual prevalence is difficult to estimate due to lack of diagnostic standardization of studies that aim to assess this parameter.^7^ Most children with CMPA develop symptoms before the age of one year, usually within a week of the introduction of cow’s milk-based formula.^8^ Some infants who are exclusively breastfed can also develop CMPA, under the hypothesis that this is due to β-lactoglobulin, a substance present in cow’s milk, which is found in breastmilk 4–6 h after the mother consumes cow’s milk.^9^, ^10^

The pathophysiological mechanism related to the development of clinical manifestations of CMPA is still being elucidated. Some manifestations are induced by IgE-mediated mechanisms, through type-1 hypersensitivity reactions, such as urticaria, angioedema and anaphylaxis. Delayed reactions are not mediated by IgE and/or are mixed immune reactions and are characterized by occurring from 24 h to weeks after the exposure. These are non-specific reactions, such as poor weight gain, vomiting, refusal to feed, and diarrhea.^11^ These characteristics, together with the absence of confirmatory laboratory tests, make the diagnostic accuracy of CMPA difficult, and some studies estimate that CMPA symptoms can be found in 5%–15% of infants overall.^9^ Therefore, when CMPA is suspected, an allergen-elimination diet should be carried out for 2–4 weeks, followed by an oral food challenge test to assess whether the symptoms are reproducible, thus confirming the diagnosis.^12^

A correct diagnosis allows an adequate diet to be given to the affected children, thus allowing favorable growth and development. On the other hand, when an elimination diet is implemented when it is not necessary, or when this diet persists, even after the child has already developed tolerance to the allergen, there may be nutritional deficits, worsening of the child and family quality of life, as well as the generation of unnecessary and significant healthcare costs.^8^, ^11^

In 2004, the correlation between food allergy and OME and ROM was postulated by James based on a series of studies that indicated allergic inflammation in the nasal mucosa as a causative factor for Eustachian tube dysfunction and subsequent OME. However, the author discusses the difficulties in obtaining confirmation of the role of food allergy in the occurrence of otitis media, since the data published at the time were limited, the assessed studies had poorly developed methodological designs and, therefore, led to overestimated and controversial results.^9^

Therefore, this study aims to review the evidence on the correlation between CMPA and ROM and OME development.

## Methods

This is a systematic review guided by the question: “Is there evidence of a correlation between CMPA and ROM and/or OME?”. The creation of the research question was structured according to the components of the PECO (Population of Interest, Exposure, Comparison and Outcome) acronym.^13^ The review included full-text studies published on any date, in English, Portuguese and Spanish, which evaluated the association between CMPA and ROM and/or OME, with a description of the parameters used for the diagnosis of these conditions. Abstracts published in conference proceedings, case reports, and studies in which the diagnoses were made by self-report or were not described were excluded. All procedures for conducting this review were performed according to the Preferred Reporting Items for Systematic Reviews and Meta-analyses (Prisma) checklist.^14^

The PROSPERO registration number is: CRD42021262210.

## Search methods for study identification

### Electronic search

The studies were identified in the Cochrane Central Register of Controlled Trials (CENTRAL), Web of Science (Clarivate Analytics), EMBASE (Elsevier), MEDLINE (PubMed), and Latin American and Caribbean Health Sciences Literature (LILACS/BIREME). The following search strategy was used in the MEDLINE database (PubMed): “Otitis Media” [MeSH] OR otitis media OR aom OR ome AND “Milk Hypersensitivity” [MeSH] OR milk proteins OR (CMA OR CMPA) OR “milk allergy” OR “milk protein allergy” OR “cow* milk protein allergy” OR allergy AND (“infant formula*” OR “milk adverse effects” OR “Food Hypersensitivity” [MeSH] OR “food allergen.” The full search strategy according to each database is detailed in Appendix 1. No search restrictions were applied.

### Other search resources

The following gray literature sources were consulted: Google Scholar, OpenGrey, Turning Research Into Practice (TRIP), and Catalogs of Theses and Dissertations from the Coordination for the Improvement of Higher Education Personnel (CAPES). The search strategy for these sources is detailed in Supplement 1. The reference lists of retrieved studies were also searched to identify other potentially eligible studies.

### Data collection and analysis

#### Study selection

In the screening stage, three authors independently evaluated the titles and abstracts of all results of the search strategy, according to the inclusion and exclusion criteria. Disagreements, when present, were resolved by a fourth reviewer. In the next step, the full text of the studies that were selected in the previous step was read independently and the studies that did not meet the inclusion criteria were excluded.

#### Data extraction and management

Data from the included studies were collected using a standardized assessment form.

### Quality assessment of included studies

**Figure 1:**
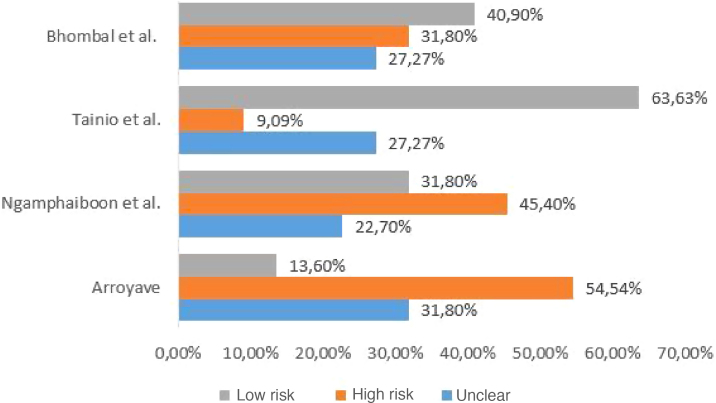
Qualitative assessment of the quality of included studies.

**Figure 2:**
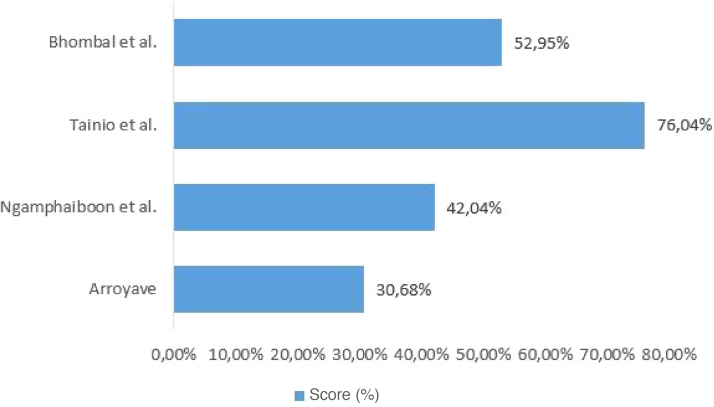
Quantitative assessment of the quality of included studies.

The checklist provided by the report prepared by the Strengthening the Reporting of Observational Studies in Epidemiology (STROBE) initiative^15^ was used to assess the quality of the included studies. Each study was independently evaluated by two reviewers and discrepancies were resolved by consensus. For data presentation, the checklist was completed in two ways. In the first, in a qualitative way, each STROBE item was assigned: “yes” (low risk of bias) when the item was fully covered, “no” (high risk of bias), when the item was not covered, or “partially”, when the item was unclear. The results were presented as shown in Fig. 1. In the second, in a quantitative way, scores were assigned to each item: 1 point for covered items; 0.5 for partially covered items and 0 for items that were not covered. For items that contained sub-items, the score was proportionally distributed, that is, in an item consisting of 5 sub-items, each sub-item received a value of 0.2. The results of this second approach are depicted in Fig. 2.

## Results

**Figure 3:**
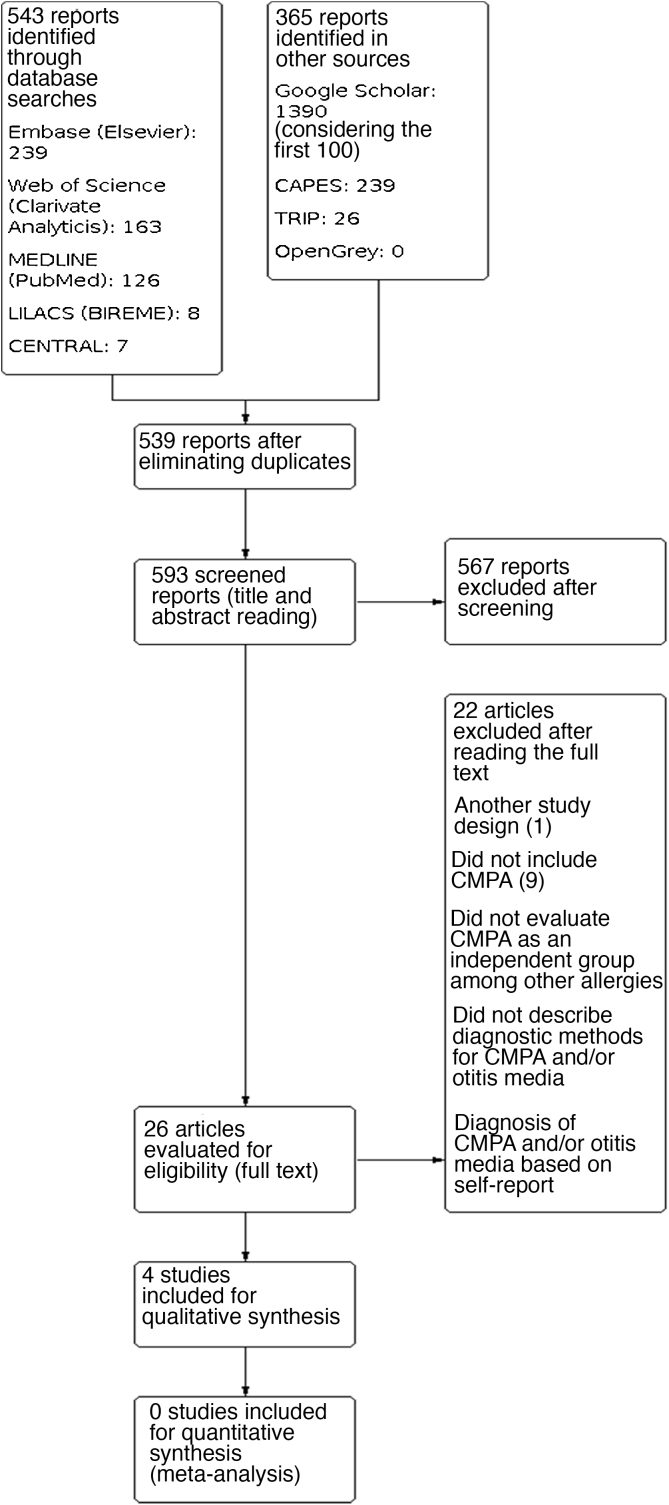
PRISMA flow diagram.

A total of 908 results were identified, and after performing the methodological steps, 4 studies were included for the qualitative synthesis. The information flow containing the phases of this review is shown in Fig. 3. We point out that only one selected study was not read in full. After contacting the journal responsible for its publication, they informed us the study is not available in digital media (Appendix 2).

The four studies included in the review evaluated the correlation between CMPA and ROM or OME. The studies included pediatric patients from different age groups, and their diagnostic criteria varied, with heterogeneity being observed. The studies were observational, of which three are cross-sectional studies and one a prospective study. The characteristics of the studies are shown in Table 1. The assessment of methodological quality is shown in Figure 1, Figure 2.

**Table 1:** Characteristics of the included studies.

Reference (author(s), year)	Place (city/country)	Study design	Characteristics of the population			Diagnostic method for diagnosing CMPA	Diagnostic method for assessing the diagnosis of otitis media	Outcomes of interest
			Sample size and profile	Gender	Age			
Tainio et al.,^19^ 1988	Helsinki, Finland	Prospective cohort	183 (children healthy at birth)	Not described.	Follow-up from birth to 2 years of age.	Repeated development of cutaneous, respiratory or gastrointestinal signs after ingestion of cow’s milk, and disappearance of these signs while on a diet other than cow’s milk; and oral food challenge test performed at the hospital.	AOM: otoscopic examination showing loss of translucency and tympanic membrane landmarks, clear inflammation or bulging, lack of mobility, or purulent discharge.	Seven children had CMPA with cutaneous manifestations during the first year of life. These tended to have otitis media more frequently during the first year when compared to children without CMPA (1.5 + 0.6 *vs*. 0.4 + 0.1; *p* < 0.1). Only two of these seven children did not have otitis media during the first year, compared to 145 of the other 176 non-allergic children (*p* < 0.001). Only one participant with CMPA had ROM.
							ROM: number of isolated episodes of otitis media occurring at the 90th percentile of the entire series of participants during the first 2 years (five episodes), or at the 95th percentile during the second year (four episodes). Isolated episodes were considered when an episode of otitis media occurred 2 months after a previous episode and after a normal otoscopic examination between the episo	
Bhombal et al.,^17^ 2006	Columbia, USA	Cross-sectional	242 (children undergoing otorhinolaryngological procedures)	Male gender: 140; female gender: 102	Age ranged from 5 months to 15 years, with a mean of 4.7 ± 2.9 years.	Measurement of total and specific IgE antibodies to milk antigens (*PharmaciaCAP*™ *IgE florescence immunoassay*).	The evaluated parameter was the performance of otorhinolaryngological procedures (bilateral myringotomy with tube insertion; bilateral myringotomy with tube insertion + adenoidectomy; adenoidectomy; or bilateral myringotomy with tube insertion + adenoidectomy + tonsillectomy).	CMPA showed a prevalence of 26 in 242 (10.7%) cases in the studied sample. In participants aged >2 years, the prevalence of CMPA was 12.9%, and in those aged <2 years, 8.5%.
Arroyave,^16^ 2001	Quéretaro, Mexico	Cross-sectional	25 (children with recurrent OME)	Male gender: 16; female gender: 9	Age ranged from 18 months to 6 years, with a mean age of 3 years.	Positive skin prick test and clinical improvement after withdrawal of the allergen.	Recurrent OME: evidence of OME lasting more than 3 months or recurrence of OME monthly in the past 6 months.	The skin prick test was positive for milk in 10 of 25 (40%) cases in the studied sample.
Ngamphaiboon et al.,^18^ 2008	Bangkok, Thailand	Cross-sectional	382 (children with CMPA)	Male gender: 214; female gender: 168	Age ranged from 7 days to 13 years, with a mean age of 14.8 months.	Significant clinical improvement after elimination of cow’s milk and symptom recurrence after reintroduction of cow’s milk. Laboratory parameters such as skin prick test or serum cow’s milk-specific IgE antibodies were only support parameters. A double-blind placebo-controlled challenge test was performed in some patients.	Diagnosis of delayed speech due to chronic OME recorded in medical records.	Delayed speech due to chronic OME was observed in 0.2% of 382 in the studied sample.

CMPA, cow’s milk protein allergy; AOM, acute otitis media; OME, otitis media with effusion; ROM, recurrent otitis media.

For the diagnosis of CMPA, two studies used clinical improvement as a parameter after withdrawal of cow’s milk from the diet and symptom recurrence after the reintroduction, one of which also considered the positive oral challenge test performed in a hospital. As for the other two studies, one of them used the measurement of total and specific antibodies to milk antigens, while the other used a positive skin prick test and clinical improvement after removal of the allergen. To identify otitis media recurrence, two studies presented the criteria used to define AOM/OME and ROM, and two studies used as parameters the recording of otorhinolaryngological surgical procedures (bilateral myringotomy with tube insertion, adenoidectomy and tonsillectomy), and the recording of the diagnosis of delayed speech due to chronic OME in medical records.

The measures of interest present in the studies and of interest for this review were: (1) prevalence of delayed speech due to chronic OME in children with CMPA; (2) prevalence of CMPA in children undergoing otorhinolaryngological procedures; (3) prevalence of CMPA in children with recurrent OME; (4) frequency of recurrent otitis media in children with CMPA and children without it. Therefore, the results found in each study were: the prevalence of delayed speech due to chronic OME was 0.2% in a sample of 382 children with CMPA; the prevalence of CMPA was 10.7% in a sample of 242 children submitted to otorhinolaryngological procedures; the prevalence of CMPA was 40% in a sample of 25 children with recurrent OME and; in a sample of 183 children followed since birth, seven children with CMPA tended to have otitis media more frequently during the first year when compared to children without CMPA (1.5 + 0.6 *vs.* 0.4 + 0.1 ; *p* < 0.1). Only one of these 7 children had ROM.

## Discussion

In recent decades, the role of allergies in the development of ROM and OME has been considerably investigated. However, when reviewing the studies evaluating the correlation between otitis media and CMPA, we observed the absence of clear case definitions, standard diagnostic criteria, or randomization and blinding to control for potential biases. The studies included in this review investigated children in mixed age groups and showed variations in the methods for the diagnosis of otitis media and CMPA.

The study developed by Arroyave^16^ evaluated the correlation between OME and food allergies. The atopy diagnosis was defined through a positive skin test and clinical improvement after removal of the allergen. When investigating multiple allergens in a sample of 25 patients, 10 (40%) patients with a positive skin test for cow’s milk antigens were identified. It is important to emphasize that this study does not clearly define the criteria used for the diagnosis of OME in the assessed population, as it only mentions the number of otitis episodes, without specifying the diagnostic criteria used to define the case. Moreover, the skin test and elimination diet used in this study as a diagnostic method for CMPA are not deemed the gold standard for considering the individual as having this food allergy.

In the analysis conducted by Bhombal et al.,^17^ the investigated parameter was the increase in total and specific IgE immunoglobulins for several food allergens, including cow’s milk, in children submitted to otorhinolaryngological procedures: bilateral myringotomy with tube insertion, adenoidectomy and tonsillectomy. A total of 242 patients were evaluated, and of these, 26 (10.7%) had an increase in specific IgE for cow’s milk when compared to the total population. However, it is known that the increase in specific IgE cannot be used as a diagnostic criterion for CMPA, but only as a complementary test that would help in the followup of patients with this type of pathology. Thus, the study concludes that although it is not possible to establish a causal relationship between food allergy and the higher frequency of these procedures, there would be some benefit in further studies on the presence of food allergy in the pediatric population with indication for these surgeries.

As for the study developed by Ngamphaiboon et al.,^18^ which aimed to investigate the most frequent clinical manifestations in children with CMPA, a delay in language development due to OME was identified in 0.2% of the 382 children included in the study. Although the study attributes speech delay to OME episodes, the existence of multiple causes for speech delay in the studied age group is emphasized; however, the study does not describe whether there are confounding risk factors. Furthermore, a blinded oral food challenge test was not performed in all children included as patients with CMPA. Therefore, the study does not show reliability to attribute a causal relationship between OME and CMPA, based on the assessed parameters.

The study by Tainio et al.^19^ aimed to investigate the risk factors related to the development of ROM during the follow-up of 198 children from birth to 2 years of age. The method used for the diagnosis of ROM was the recording of five episodes of otitis media confirmed by otoscopy in the first year of life or four episodes in the second year of life, and CMPA was confirmed through an oral food challenge test. Of the evaluated children, only one patient with CMPA developed ROM. Thus, the study demonstrated that CMPA cannot be considered a risk factor for the diagnosis of ROM. Furthermore, although serum IgG immunoglobulin levels for cow’s milk in children with ROM are higher than in the other children in the studies, it is known that such evidence has no clinical significance, that is, it does not provide a diagnosis of allergy.

When investigating the relationship between CMPA and AOM, only Tainio et al. considered the concurrence of well-established risk factors for AOM, such as parental smoking and daycare attendance, and protective factors such as breastfeeding duration. The absence of a description of these factors in similar studies demonstrates the lack of strategies to control confounding factors. The quality assessment of the included studies varied between 30.68% and 76.04% (of a total of 100%). Thus, the results found in the studies included in this review do not show methodological rigor and cannot be generalized.

Finally, we emphasize that published meta-analyses on risk factors for the development of otitis media indicate passive smoking, use of pacifiers, day care attendance, and lack of breastfeeding as reliable factors.^20^, ^21^ As for atopies or allergies, evidence suggests that respiratory allergies, such as allergic rhinitis, may contribute to the onset of recurrent otitis media.^21^ Nevertheless, regarding the demonstration of a correlation between CMPA and ROM/OME, we emphasize that further studies are still needed.

## Conclusions

Observational studies were identified, which showed considerable heterogeneity regarding the methodological design, age group of the studied population and diagnostic methods for CMPA and otitis media. Thus, the results found in this review do not allow us to state that there is a correlation between CMPA and the development of ROM or OME. It is necessary to develop studies with adequate methodological designs and standardize the diagnostic criteria.

## Conflicts of interest

The authors declare no conflicts of interest.
