# Supplementary material for: Correlation between cow’s milk protein allergy and otitis media: a systematic review
Source: Braz J Otorhinolaryngol. 2021 Oct 18;88(5):803–11. doi: 10.1016/j.bjorl.2021.07.005 (PMC9483997; doi:10.1016/j.bjorl.2021.07.005)

BJORL-D-21-00235 – Supplementary Material

**Appendix 1** Search strategies.

| **Databases** | |
| --- | --- |
| **MEDLINE (PubMed)** | “Otitis Media”[MeSH] OR otitis media OR aom OR ome AND “Milk Hypersensitivity” [Mesh] OR milk proteins OR (CMA OR CMPA) OR “milk allergy” OR “milk protein allergy” OR “cow* milk protein allergy” OR allergy AND (“infant formula*” OR “milk adverse effects” OR “Food Hypersensitivity” [MeSH] OR “food allergen” |
| **EMBASE (Elsevier)** | ‘otitis media’/exp AND ‘milk allergy’/exp |
| **Web of Science (Clarivate Analytics)** | ‘otitis media’ OR aom OR ome AND “milk hypersensitivity” OR milk proteins OR (CMA OR CMPA) OR “milk allergy” OR “milk protein allergy” OR “cow* milk protein allergy” OR allergy AND (“infant formula*” OR “milk adverse effects” OR “food hypersensitivity” OR “food allergen”. |
| **LILACS (BIREME)** | MH: Otitis Media OR MH:C09.218.705.663$ OR “otitis media” OR “Otite Média” AND “Milk Hypersensitivity” OR “Hipersensibilidad a la Leche” OR “Hipersensibilidade ao Leite” OR “Food Hypersensitivity” OR “Hipersensibilidad a los Alimentos” OR “Hipersensibilidade Alimentar” |
| **CENTRAL** | #1 MeSH descriptor “Otitis Media” explode all trees |
|  | #2 otitis OR aom OR ome AND MeSH descriptor “Milk Hypersensitivity” explode all trees |
| **Gray literature** | |
| **Google Scholar** | “otitis media” AND “milk allergy” OR “milk hypersensitivity” |
| **OpenGrey** | “otitis media” AND “milk allergy” OR “milk hypersensitivity” |
| **Turning Research Into Practice (TRIP)** | “otitis media” AND “milk allergy” OR “milk hypersensitivity” |
| **Catalog of theses and dissertations (CAPES)** | “otitis media” AND “milk allergy” OR “milk hypersensitivity” OR “alergia ao leite de vaca” |

**Appendix 2** Requisition for a potentially eligible article unavailable for access.


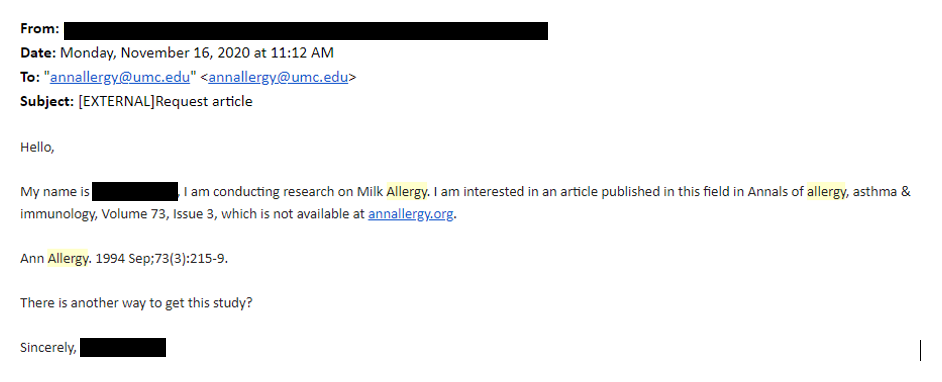


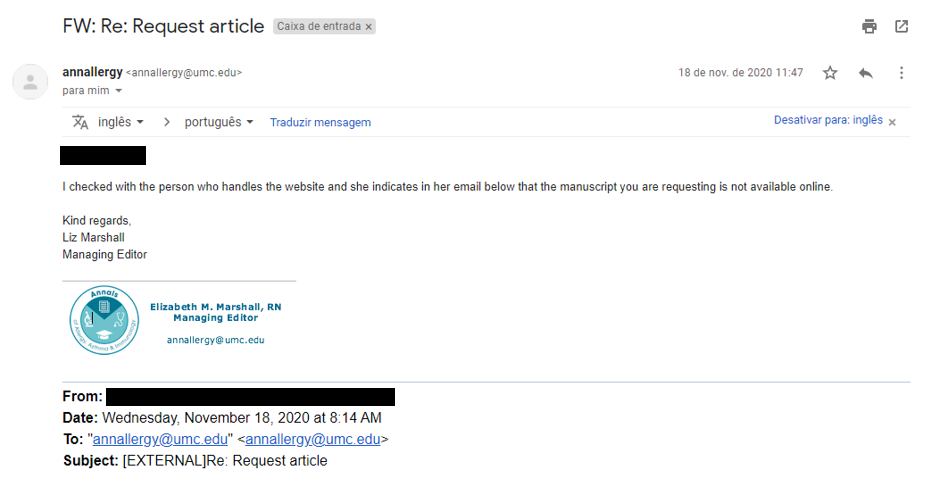

Supplement: Supplementary file 1 [file mmc1.docx]
